# Response of Two Mytilids to a Heatwave: The Complex Interplay of Physiology, Behaviour and Ecological Interactions

**DOI:** 10.1371/journal.pone.0164330

**Published:** 2016-10-13

**Authors:** Celia Olabarria, Ignacio Gestoso, Fernando P. Lima, Elsa Vázquez, Luc A. Comeau, Filipa Gomes, Rui Seabra, José M. F. Babarro

**Affiliations:** 1 Departamento de Ecoloxía y Bioloxía Animal, Universidade de Vigo, 36310, Vigo, Spain; 2 MARE – Marine and Environmental Sciences Centre, Quinta do Lorde Marina, Sítio da Piedade, 9200-044, Caniçal, Madeira, Portugal; 3 CIBIO, Centro de Investigação em Biodiversidade e Recursos Genéticos, Universidade do Porto, Campus Agrário de Vairão, 4485-661, Vairão, Portugal; 4 Department of Fisheries and Oceans, Science Branch, Gulf Fisheries Centre, P.O. Box 5030, Moncton, NB, E1C 9B6, Canada; 5 Department of Biotechnology and Aquaculture, Instituto de Investigaciones Marinas CSIC, Eduardo Cabello 6, 36208, Vigo, Spain; Fred Hutchinson Cancer Research Center, UNITED STATES

## Abstract

Different combinations of behavioural and physiological responses may play a crucial role in the ecological success of species, notably in the context of biological invasions. The invasive mussel *Xenostrobus securis* has successfully colonised the inner part of the Galician Rias Baixas (NW Spain), where it co-occurs with the commercially-important mussel *Mytilus galloprovincialis*. This study investigated the effect of a heatwave on the physiological and behavioural responses in monospecific or mixed aggregations of these species. In a mesocosm experiment, mussels were exposed to simulated tidal cycles and similar temperature conditions to those experienced in the field during a heat-wave that occurred in the summer of 2013, when field robo-mussels registered temperatures up to 44.5°C at low tide. The overall responses to stress differed markedly between the two species. In monospecific aggregations *M*. *galloprovincialis* was more vulnerable than *X*. *securis* to heat exposure during emersion. However, in mixed aggregations, the presence of the invader was associated with lower mortality in *M*. *galloprovincialis*. The greater sensitivity of *M*. *galloprovincialis* to heat exposure was reflected in a higher mortality level, greater induction of Hsp70 protein and higher rates of respiration and gaping activity, which were accompanied by a lower heart rate (bradycardia). The findings show that the invader enhanced the physiological performance of *M*. *galloprovincialis*, highlighting the importance of species interactions in regulating responses to environmental stress. Understanding the complex interactions between ecological factors and physiological and behavioural responses of closely-related species is essential for predicting the impacts of invasions in the context of future climate change.

## Introduction

Climate change is likely to alter community composition in many terrestrial and marine systems through range shifts, differential responses to thermal stress, and changes in interactions between species [1,2]. Organisms inhabiting the rocky intertidal zone are especially vulnerable to climate change because they are exposed to both marine and terrestrial conditions, and some species are sometimes exposed to conditions close to their physiological limits for stress [3]. In this zone, abiotic conditions may also shift even more dramatically over scales of just a few metres.

Temperature is one of the most important abiotic factors affecting the distribution and physiological performance of organisms in the intertidal zone (e.g. [4–10]). Extreme events, such as heatwaves, are predicted to increase in severity and frequency as a consequence of climate change [11] and will probably affect intertidal systems [12]. Until recently, many studies of the thermal physiology of intertidal organisms and the mechanism involved in temperature sensitivity have focused on temperature stress during immersion, even though intertidal organisms typically experience thermal stress during emersion. During emersion, organisms such as mussels and limpets are exposed to rapidly fluctuating and often extreme temperatures; their body temperatures regularly exceed the water temperature by more than 15°C [4,13,14] and also frequently exceed the temperature of the surrounding air, approaching sub-lethal limits [10,15,16]. Thermal stress can have significant physiological consequences with profound implications for ecological interactions [9]. For example, competitive interactions between barnacle species can change under heat and/or desiccation stress conditions [6], and high temperatures may exert stronger effects on top predators than on their prey [17]. Life in the intertidal zone is therefore often associated with adaptive responses such as gregarious behaviour, increased thermal resistance, thermal stability of key metabolic enzymes, reduced evaporation, and stress-induced expression of heat shock proteins [18–21]. Although various studies have documented the consequences of the intertidal stress gradient on invertebrates (e.g. [14,19,22–24]), the sub-lethal effects of stress on the ecology and physiology of these organisms have been less well studied.

Mussels are dominant competitors for space on many temperate rocky shores throughout the world [17]. They exhibit gregarious behaviour, which is considered an advantageous evolutionary strategy that provides protection from predators or harsh environmental conditions, and favours reproductive success [25,26]. For life in the high or mid intertidal zone, thermal buffering is one advantage provided by living in aggregations with con-specifics or heterospecifics [26]. However, as well as benefits there are also some costs (such as increased competition for resources) associated with living in aggregations. In some cases, the outcome of the interaction will depend on the spatial position of individuals within the aggregation [8,27]. For example, although specimens of the blue mussel *Mytilus edulis* (Linnaeus 1758) living at the edge of a dense aggregation face higher predation rates than individuals located near the centre of the aggregation, their growth rate is 50% higher because of greater access to food resources [27]. Moreover, mussels at the centre of an aggregation are often exposed to higher temperatures [28]. Morphological features, i.e. body size, shell thickness/size, and behaviour of individuals, may affect the outcome of such interactions [24,29]. Nicastro and collaborators [24] found that under conditions of heat stress, aggregations of the gaping mussel *Perna perna* (Linnaeus 1758) exhibited lower mortality rates than aggregations of the non-gaping mussel *Mytilus galloprovincialis* (Lamarck 1819) because gaping behaviour of *P*. *perna* ameliorated stressful environmental conditions of mussels through evaporative cooling.

The black pygmy mussel *Xenostrobus securis* (Lamarck 1819), an invasive species endemic to the brackish waters of New Zealand and Australia, potentially has negative impacts on other ecosystems [30]. It has been recorded as an invasive species along the coast of the Mediterranean Sea and in Japanese waters [31–33]. It has also spread to the inner part of the Galician Rias Baixas (NW Spain) [34], where it co-occurs with the commercially-important mussel *M*. *galloprovincialis*, forming monospecific and mixed patchy aggregations of differing densities on diverse substrates [35]. In this environment, facilitative rather than competitive interactions between juveniles of the two species occur, although the interactions vary depending on the environmental context [36]. The species differ in morphological aspects [36,37], but also in physiological and behavioural traits. For example, *X*. *securis* shows greater morphological plasticity of byssal threads than *M*. *galloprovincialis* when exposed to stressful environmental conditions [38]. In mixed beds, the species tend to distribute in two layers with *M*. *galloprovincialis* migrating to the top [39]. In contrast to *M*. *galloprovincialis* [24], *X*. *securis* is a gaping species, i.e. it opens and closes its shell during emersion [40]. Non-gaping behaviour reduces water loss at the cost of less efficient use of stored energy because animals must rely on anaerobic metabolism [41]. This important difference in behaviour thus potentially determines the relative performance of the two species when exposed to extreme temperatures during low tide, e.g. during heatwaves. Indeed, the relative performance of the species under a range of environmental conditions determines the outcome of interactions and ultimately the invasion success of *X*. *securis* [36].

We conducted a short-term mesocosm experiment to evaluate the effect of a simulated heatwave on the physiological performance of the two species during both emersion and immersion cycles. We measured lethal and sub-lethal responses of mussels in monospecific and mixed species aggregations. The response variables used to quantify the accumulated effects of lethal and sub-lethal stress were mortality, water loss, heat shock protein level (Hsp70), respiration rate, gaping behaviour and heart rate. Specifically, we tested the hypothesis that the invasive species *X*. *securis* is more resistant and resilient to heatwave stress than *M*. *galloprovincialis*. We also tested whether the response of each species to heatwave-induced stress is influenced by species interactions, i.e. the type of aggregation. We discuss how variations in the responses of closely related mussel species may have important implications for ecological processes in intertidal communities under both current and future environmental scenarios.

## Materials and Methods

### Ethics Statement

Permission and ethical approval were not required as the study did not involve endangered or protected species. Mesocosms were designed as closed systems to prevent escape of non-indigenous species. Sampling of organisms was arranged jointly by the University of Vigo and Xunta de Galicia and complied with all relevant regulations.

### Heatwave conditions

To record temperatures in the field, we used robo-mussels that mimic the thermal characteristics of living mussels [9]. These were made by placing a temperature logger (DS1922L iButton^®^) inside two empty mussel valves (size ~ 30 mm), which were then filled with silicone sealant and left to dry at air temperature for 48 h. The robo-mussels were attached to the substratum with marine grade epoxy resin (Splash zone, A788), in an approximate growth position in the middle of small beds and were left in the field between 1 July to 28 August 2013. Two exposed wires protruding from the shell served as contacts for logger programming and subsequent data retrieval (see [42]). Loggers were programmed to record data at 30-min intervals with a resolution of 0.5°C. Robo-mussels were tested in pairs by placing them next to live mussels fitted with thermocouple probes. Test temperatures ranged from 16°C (during submersion by high tide) to 45°C (during daytime emersion). Readings from robo-mussels and live animals were strongly correlated (Pearson’s correlation coefficient of 0.983, p< 0.05), with a low average bias (-0.36°C) and a small root mean square error (1.81°C), especially considering the range of temperatures used in the main experiment. Readings were also consistent among robo-mussels (standard deviation of 0.54°C), which is lower than the standard deviation for live animals (1.22°C).

The temperature treatments used in the mesocosm experiment were designed to mimic a heatwave that occurred between 5 and 10 July 2013, when field robo-mussels registered temperatures up to 44.5°C during low tide (S1 Fig).

### Collection and acclimation of mussels

Individual specimens of *M*. *galloprovincialis* and *X*. *securis* were collected from the inner part of the Ria de Vigo (NW Spain), at Cesantes (42°19'20.86"N; 8°36'57.99"W). Similar-sized individuals (28–30 mm long) were collected from the mid-shore and taken to the laboratory. After biofouling was stripped from the shells and the byssus was removed from the ventral margin, individuals were assembled in aggregations and allowed to establish primary attachment to experimental units, i.e. a biodegradable mesh on PVC plates. Two types of aggregation were prepared for each species: monospecific aggregations (30 individuals) and mixed aggregations (15 individuals of each species). Prior to the experiment, plates with aggregations were held in the experimental mesocosms for 4 d under stable high tide conditions (20 ± 0.2°C, 35 ± 0.5 psu and 12 h: 12 h light: dark photoperiod), consistent with the collection site. The seawater was changed twice a day during this period, and ammonium levels were checked daily. Mussels were then acclimated for 5 d to laboratory-simulated tidal cycles (2 low and 2 high tides), corresponding to the typical semidiurnal tidal regime in the study area (see [36]). Environmental conditions were similar to those experienced in the earlier 4-d period, except that mussels were exposed to an air temperature of 30 ± 0.4°C during emersion. During the acclimation and the experimental period, mussels were fed a mixed diet, specifically a ration of 3% of total tissue dry weight supplied in a single dose every 2 d.

### Experimental set-up

**Fig 1:**
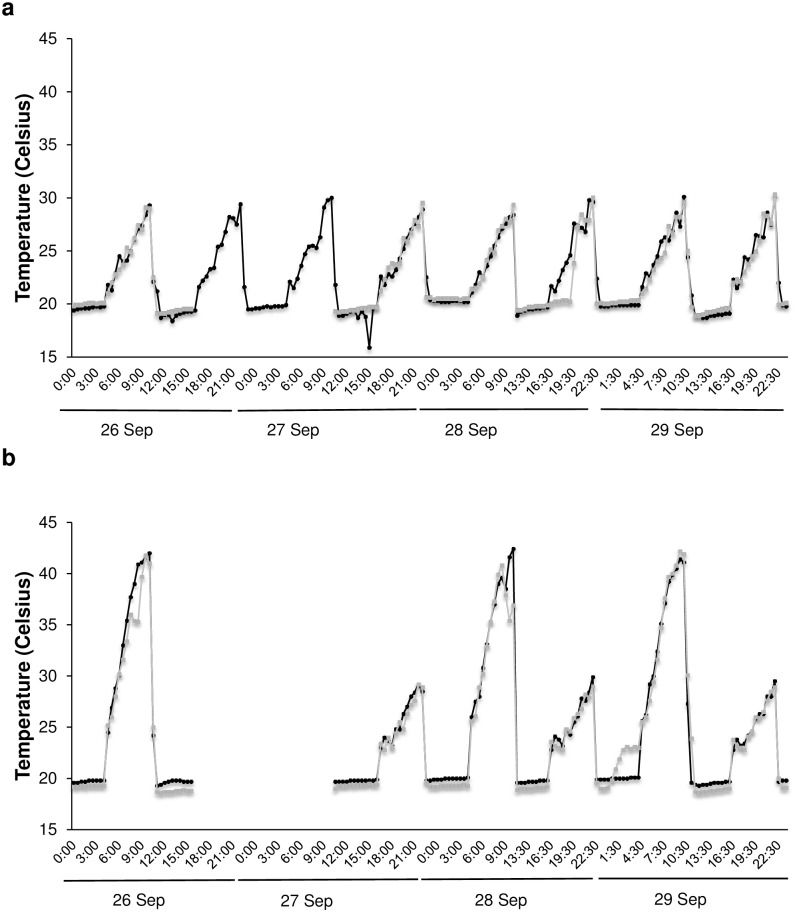
Temperature profiles in the mesocosms. Temperature profiles in the four experimental tanks (A-D): a) Control conditions (tank A: black line; tank B: grey line); b) Heatwave conditions (tank C: black line; tank D: grey line). Missing values between 1600h on 26 September and 1000h on 27 September are due to a failure in the recording system.

After acclimation of the mussels for 9 d, the experiment was conducted, between 26 and 29 September 2013, in an isothermal walk-in chamber held at a temperature of 20 ± 0.2°C. The seawater temperature during high tides was consistent with the temperature in the isothermal chamber. To produce temperature profiles similar to those experienced by mussels at low tide in the field, the air temperature during low tide was increased by using heating infrared lamps (150 W, Exo Terra) positioned over the experimental mesocosms (350-L PVC tanks). The air temperature was regulated by digital temperature controllers (Aqua Medic^®^ AT Control System controllers, GmbH, Bissendorf, Germany) and recorded via the individual temperature probes inserted in the robo-mussels. This system enabled continuous control and recording of air temperature with an error of 0.2°C. Control and heatwave treatments were assigned at random to each experimental tank (i.e. two tanks for each experimental temperature), and the heatwave treatment was applied during morning low tide. Use of digital controllers allowed us to ramp the temperature smoothly from the initial temperature at high tide (20°C) to the desired experimental temperatures (29 and 42°C) in the control and heatwave treatments at morning low tide and to 29°C in both treatments during afternoon low tide. The ramp-up durations was 6 h, i.e. with a constant increase of 1.5°C h^-1^, or 5°C h^-1^ with an increase of 2°C in the last 2 h, for the control and heatwave treatments respectively (Fig 1). Four experimental units of each aggregation were placed at a random position in each experimental tank and covered with an extensive mat of *Fucus vesiculosus* (Linnaeus 1753), which formed a natural cover during low tide. The relative humidity in the tanks was monitored with a humidity logger (DS1923 iButton^®^) placed underneath the macroalgal canopy during low tide on two consecutive days. During emersion, the relative humidity always exceeded 79% in all experimental tanks.

### Response variables

#### Mortality

We assessed mortality immediately after the 4 days of the experimental exposure period by recording the number of dead mussels in each plate (i.e. experimental unit) on the last day of the experiment.

#### Water loss

On the last day of the experiment, ten mussels of each species from monospecific aggregations were exposed to control and heatwave treatments for 6 h. Individuals were weighed (± 0.01 g) at the beginning of the experiment and every 20 min thereafter to monitor water loss. At the end of the experiment, each individual was dried to constant weight at 60°C to determine the dry weight (tissue and shell). The values thus obtained were used to calculate the percentage of water loss.

#### Heat shock proteins (Hsp70)

At the end of the experiment, mussel mantle tissue was collected from 4 individuals of each species and aggregation exposed to the two temperature treatments and to two different types of tidal exposure: (i) after 6-h emersion (hereafter referred to as emersion), and (ii) after immersion for 1.5-h following exposure to either heatwave or control emersion conditions (hereafter referred to as re-immersion). Re-immersion measurements allowed us to evaluate the recovery of mussels after emersion conditions.

Samples were immediately frozen in liquid nitrogen on collection and were maintained at -80°C until further processing. Approximately 20 mg of tissue was placed in a Tris-sodium dodecyl sulphate (SDS) buffer [0.4% Tris-HCl pH 6.8, 2% sodium dodecyl sulphate (SDS), 1% ethylenediaminetetraacetic acid (EDTA), 1% Protease inhibitor cocktail (Thermo Scentific# 78444, IL, USA)]. Samples were heated at 100°C for 5 min and homogeneized twice at 30 Hz for 5 min and then centrifuged at 15800 *g* for 15 min. The supernatant was decanted, stored at -80°C and used for subsequent analyses. The protein concentrations in the samples were determined using the BCA protein assay reagent (Pierce, IL, USA). Similar amounts (mass-wise) of sample were subsequently loaded onto the gels.

The samples were boiled at 100°C for 5 min and then mixed 1:1 (v/v) with Laemmli sample buffer (Sigma-Aldrich #S3401, MO, USA) plus 5% 2-mercaptoethanol). Proteins were separated by electrophoresis on small format SDS-polyacrylamide gels in Mini-PROTEAN Tetra Cell cast systems, BioRad, CA, USA (4% for stacking gels and 7.5% for the resolving gels, for optimal protein resolution). For quantitative comparison of different gels, a “common sample” was prepared by mixing aliquots of several homogenates (total 1 ml). In each gel, two lanes were loaded with 15 μg of “common sample”, two “standard” lanes were loaded with 80 ng of purified recombinant human HSP70B’ protein (#ADI-SPP-762, Enzo Life Sciences, NY, USA), one lane was loaded with 5 μl of pre-stained markers (#161–0374, BioRad, CA, USA), and the remaining eight lanes were loaded with the samples to be quantified (15 μg each). Gels were run at 100 V for 20 min followed by 120 V for 65 min in running buffer (1.4% glycine, 0.3% Tris-base, 0.1% SDS, pH 8.3).

Proteins were transferred to 0.45-μm polyvinylidene difluoride (PVDF) membranes (Millipore Immobilon #IPVH00010, Fisher Scientific, MA, USA) in transfer buffer (0.06% Tris-base, 0.03% glycine, 20% methanol), at 100 V for 35 min in a Mini Trans-Blot Electrophoretic Transfer Cell (#170–3930, BioRad, CA, USA). Western blotting was performed on the PVDF membranes according to Tomanek and Sanford [19]. Membranes were incubated with monoclonal rat antibody (IgG) against Hsp70 (MA3-001; Affinity Bioreagent, Golden, CO). The membranes were washed before being incubated for 30 min with a rabbit anti- rat bridging antibody (IgG) solution (1:2000 dilution in BA; Vector, AI-4000) and then washed again several times. Finally, the membranes were incubated with a horseradish-peroxidase protein A solution (1:5000 dilution in BA; Bio-Rad) for 30 min. Bands were visualized using a chemiluminescence detection method and the blots were then exposed to X-ray film. Film images were scanned on a densitometer and the digitized images were analysed with Image J v1.4 image analysis software. Here, we report the combined quantity of both the constitutive and inducible forms of Hsp70 because they could not be reliably distinguished. However, quantification of total Hsp70 levels is considered even more informative from an ecological point of view because it summarizes the ability of organism to cope with thermal stress [43,44]. All samples were run three times, i.e. we performed three technical replicates. The data analysed comprised the mean values from the three runs.

Quantitative western blotting across gels is particularly affected by variations in the efficiency of protein transfer and binding to the blotting membrane, and by irregularities in the preparation and pipetting of samples [45]. We therefore followed the standardization procedure described in Lima et al. [21]. In each gel, we first quantified the optical density of the “common sample” bands relative to the average optical density of the bands in the two “standard” lanes (those with a known amount of purified HSP70B’). We then normalized the optical density of the Hsp70 by the optical density of a housekeeping protein band (ɑ-tubulin). Finally, we quantified these normalized values relative to the optical density of the “common sample” bands that had previously been assessed for each gel.

#### Respiration

Respiration rates of mussels were estimated by measuring oxygen fluxes over 20 min in 30-ml closed respirometry chambers. Oxygen consumption by 4 mussels of each species and aggregation and by two controls (i.e. seawater with two empty valves) was measured simultaneously for each different temperature treatment and tidal exposure (see above, section Hsp70): (i) emersion and (ii) re-immersion. All measurements were conducted at constant temperature (20°C), and movement of seawater in the respirometry chambers was maintained by placing the chambers on a magnetic stirrer. The water inside the chambers was renewed before each new incubation. The oxygen-saturation level in the chambers never fell below 50%.

Dissolved oxygen concentration and temperature inside the incubation chambers were measured every 60 sec via a luminescent dissolved oxygen (LDO) probe connected to a portable oxygen meter (Hach^®^ HQ40). Samples were incubated during daylight hours and all measurements were made within a period of 6 h. Respiration rate was estimated by regressing oxygen concentration (mg L^-1^) in the chamber over time, and it was expressed as μmol O_2_ g^-1^ h^-1^ (i.e. oxygen consumption). Estimates were normalized by the volume of seawater inside the chamber and mussel biomass (tissue dry weight). Additionally, controls served as blanks to correct for respiration rates of bacteria and zooplankton.

#### Gaping behaviour

Valve opening was monitored continuously during the experimental period with a valvometry system (see [46] for a detailed description). A coated Hall element sensor (HW-300a, Asahi Kasei, Japan) was glued to one valve at the maximum distance from the hinge. A small magnet was then glued to the other valve, directly below the Hall sensor. The magnetic field between the sensor and magnet depended on the gap between the two valves. The magnetic field in the form of output voltage (lV) was acquired by strain recording devices (DC 104R, Tokyo Sokki Kenkyujo Co., Japan). Output voltage was recorded every 5 min and was subsequently converted into valve opening by applying conversion algorithms specific to each sensor assembly.

At the end of the experiment, the adductor muscle was severed, and small calibration wedges were placed between the two valves at the point farthest from the hinge. Wedge height was 1–6 mm. The voltage and wedge height (i.e. valve opening) were strongly correlated (r^2^ > 0.90). Valve-opening (mm) data were converted into gape angles (θ in degrees) by using the following equation [47]:

$$\theta \;=\;2\;arcsin\;\left(\frac{0.5\;W}{L}\right)\;\times \;100$$

where W is the distance that the valve opens (mm) and L is the length of the mussel shell (mm).

Although temperature is known to influence Hall measurements, laboratory trials revealed that changes in gaping due to temperature were small (<0.13°) within the context of the study.

Five mussels of each species (2 and 3 mussels from different monospecific and mixed aggregations, respectively) were logged in each experimental tank, i.e. ten mussels per tank.

#### Heart rate

Heart rate was determined using a non-invasive IR-sensor technique, which has been successfully used with diverse intertidal organisms (see [48] for a detailed description). Basically, the technique combines an IR emitter and an IR detector in a small package. Gluing the sensor to the shell of a mussel, above its heart, allows IR light to pass through the shell and illuminate the heart and nearby circulatory vessels. Changes in shape or volume of the circulatory structures during a heartbeat, cause a change in the amount of IR light reflected from the mussel back to the IR detector. These changes in reflected IR light, transduced to changes in electrical current, are then electronically amplified and filtered, and the signal is logged into a memory card by a microprocessor (see [49] for a detailed description of the equipment). Data were recorded continuously for 1 min every 15 min throughout the experimental period. In each experimental tank, 4 mussels of each species were monitored (2 mussels for each type of aggregation), i.e. eight mussels per tank. The heart rate was computed automatically using a custom-made R (R citation) script.

### Data analysis

We fitted Generalized Linear Mixed Models for all the response variables, with Tank as a random factor nested within Temperature treatment. Analysis of gaping behaviour (i.e. shell gape angle and occurrence of valve opening) and heart rate included an additional random effect for individuals to account for the repeated measures within individuals. For all response variables except mortality rate, these models showed no variance associated with Tank (estimates of 0). Thus only changes in mortality were evaluated using a Generalized Linear Mixed Model (GLMM), and simpler GLMMs were used for the remaining variables. Unfortunately, the data did not allow use of a full model for mortality because the three-way interaction yielded unstable estimates and this term had to be removed. Changes in water loss, Hsp70 and respiration were tested using Generalized Linear Models (GLMs) and changes in gaping behaviour and heart rate were analysed using Generalized Estimating Equations (GEEs), an extension of GLMs for correlated data [50]. GEEs were useful in this study for two reasons. First, our hypothesis applied to the entire duration of the experiment and not to specific experimental days. Second, as each logged mussel was monitored continuously, correlations between observations over time for the same individual had to be taken into consideration.

The type of model and canonical link function used varied depending on the response variable analysed. Mortality was modelled as the proportion of dead individuals per experimental unit, using a log-linear model, with Temperature (HW: heatwave; nHW: control conditions) and Aggregation (Myt-alone: *M*. *galloprovincialis* in a monospecic aggregation; Myt-mixed: *M*. *galloprovincialis* in a mixed aggregation, Xen-alone: *X*. *securis* in a monospecific aggregation and Xen-mixed: *X*. *securis* in a mixed aggregation) as fixed factors, and Tank as a random factor nested within Temperature. In this case, we assumed a binomial distribution of the error term and used a logit link. Water loss data were analysed using a model with Temperature (HW and nHW) and Aggregation (Myt-alone and Xen-alone) as fixed factors. A Gamma distribution of the error term was assumed and a log link was used. In the case of Hsp70 and respiration, i.e. absolute values, we used models with Temperature (HW and nHW), Tidal exposure (emersion and re-immersion) and Aggregation (Myt-alone, Myt-mixed, Xen-alone and Xen-mixed) as fixed factors. In both cases, we assumed Gaussian distributions of the error terms and used an identity link.

Shell gape angle, gaping occurrence and heart rate data were analysed by GEE models, with Temperature (HW and nHW), Tidal exposure (emersion and immersion) and Aggregation (Myt-alone, Myt-mixed, Xen-alone and Xen-mixed) as fixed factors. For shell gape angle and heart rate, Gaussian distributions of error terms were assumed and an identity link was applied. For gaping occurrence a binomial distribution of the error term was assumed and a logit link applied. A first order autoregressive model, AR(1), was specified in all analyses by assuming a time dependence for each mussel. Model residuals were plotted against fitted values for model validation [51].

Shell gape angle, gaping occurrence and heart rate were analysed for each mussel and averaged at 5- and 15-min intervals (gaping behaviour and hear rate, respectively) over the 6-h tidal cycle under both immersed and emerged conditions on each day of the experimental period. The heart rate data were normalized by dividing the average heart rate of each mussel per tidal cycle and day of the experimental period by the basal heart rate of each individual. The basal rate was calculated as the mean heart rate of each individual during both immersion and emersion over 2 d prior to the start of the experiment. The number of replicates analysed was smaller than the initial number because some of the mussels logged for gaping behaviour died and the heart rates of individuals from one control tank were not recorded due to logger failure.

The GLMM was constructed using the glmer function of the *lme4* package. GLMs were constructed using the glm function of the *stats* package and GEEs were constructed using geeglm functions of the *geepack* package in the public-domain software, R 3.1.2 [52]. All data are reported as means ± S.E.

## Results

### Mortality and water loss

**Fig 2:**
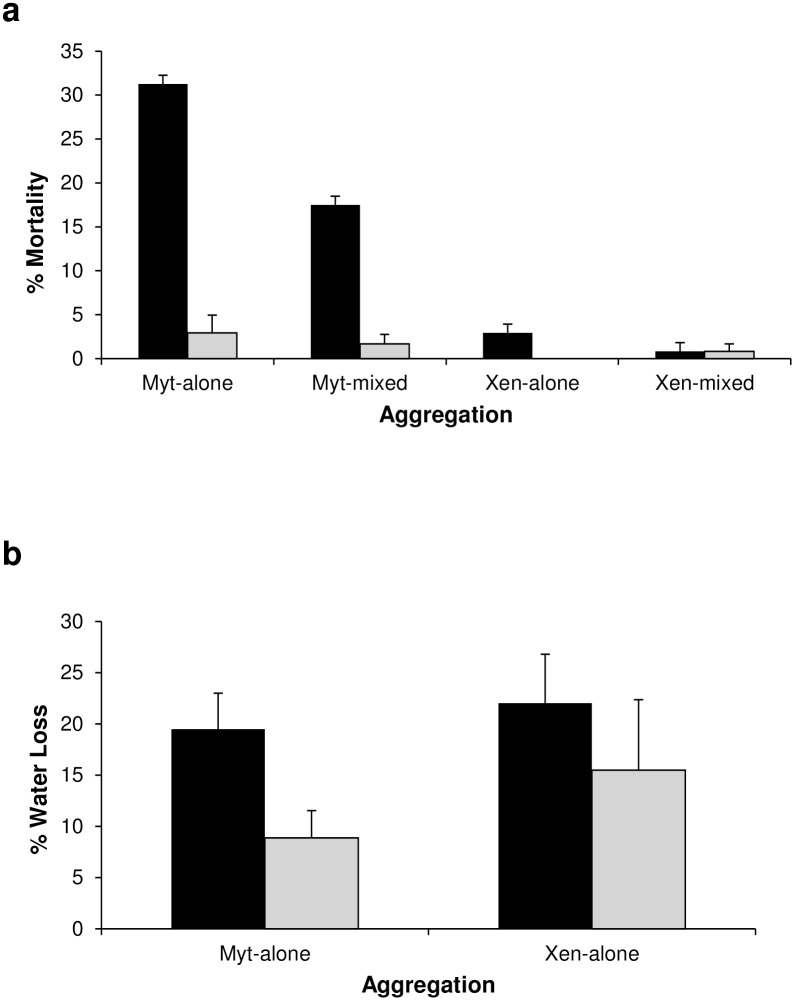
Mortality and water loss of mussels in different temperature treatments and aggregations. Mean (+ SE) values of a) % mortality (n = 8) and b) % water loss (n = 6 to 10) in the two temperature treatments (HW: heatwave in black; nHW: control in grey) and in the aggregations (Myt-alone: monospecific *M*. *galloprovincialis*; Myt-mixed: *M*. *galloprovincialis* in mixed aggregation with *X*. *securis*, Xen-alone: monospecific *X*. *securis*; Xen-mixed: *X*. *securis* in mixed aggregation with *M*. *galloprovincialis*). Mortality data were pooled across tanks.

Mortality differed significantly between temperature treatments (p = 0.05, Table 1) and aggregations (p<0.001, Table 1). Mortality was higher in individuals exposed to heatwave conditions than in controls (Fig 2a). Mortality was highest in *M*. *galloprovincialis*, especially in monospecific aggregations in which mortality was twice that of mixed aggregations, while only a few individuals of *X*. *securis* died, irrespective of the aggregation (Fig 2a).

**Table 1:** Summary of results of GLMM testing the effect of experimental treatments on mortality.

	df	F	p	Variance	SD
*Mortality*					
Random effect					
Temperature: Tank				0.22	0.46
Fixed effects					
**Temperature**	1, 2	18.42	**0.050**		
**Aggregation**	3, 57	22.49	**0.000**		

The fixed factors included in the model were Temperature (Te) and Aggregation (Agg), and the random factor was Tank nested within Temperature. The Te factor included two levels (HW and nHW), the Agg factor included four levels (Myt-alone, Myt-mixed, Xen-alone and Xen-mixed), and the Tank factor included two levels. Four replicates were considered in the analysis.

After emersion for 6 h, *X*. *securis* and *M*. *galloprovincialis* lost on average 19% and 15% of total body water respectively, although the difference was not significant (Table 2). The response of individuals within species was highly variable (Fig 2b). Temperature had a significant effect on rates of water loss, and water loss was higher under heatwave than control conditions (p< 0.05, Table 2). Mussels lost on average 20% and 12% of their total body water under heatwave and control conditions, respectively (Fig 2b).

**Table 2:** Summary of results of GLMs testing the effects of experimental treatments on water loss, heat shock proteins and respiration.

	df	Deviance	Resid. df	Resid. Deviance	p
*Water loss*					
Null			32	22.79	
**Temperature (Te)**	1	2.66	30	19.52	**0.035**
Aggregation (Agg)	1	0.61	31	22.18	0.313
Te x Agg	1	0.37	29	19.14	0.429
*Hsp70*					
Null			62	157.28	
**Temperature (Te)**	1	9.81	58	127.07	**0.011**
**Tidal exposure (Ti)**	1	9.44	57	117.63	**0.012**
**Aggregation (Agg)**	3	20.39	59	136.89	**0.003**
Te x Ti	1	4.65	50	90.74	0.079
**Te x Agg**	3	13.96	54	103.67	**0.026**
Ti x Agg	3	8.28	51	95.38	0.139
**Te x Ti x Agg**	3	19.78	47	70.96	**0.004**
*Respiration*					
Null			63	6558.70	
**Temperature (Te)**	1	761.19	59	4949.00	**0.000**
Tidal exposure (Ti)	1	185.66	58	4763.30	0.092
**Aggregation (Agg)**	3	848.54	60	5710.20	**0.004**
Te x Ti	1	196.42	51	3706.70	0.084
Te x Agg	3	132.52	55	4630.80	0.568
**Ti x Agg**	3	727.64	52	3903.20	**0.011**
**Te x Ti x Agg**	3	556.44	48	3150.30	**0.030**

The fixed factors included in the models, i.e. Temperature (Te), Tidal exposure (Ti) and Aggregation (Agg), varied depending on the response variable analysed. The Te factor included two levels (HW and nHW), the Ti factor included two levels (emersion and re-immersion), and the Agg factor included either 2 (Myt-alone and Xen-alone) or 4 levels (Myt-alone, Myt-mixed, Xen-alone and Xen-mixed). Absolute values of respiration were used. The number of replicates varied depending on the response variable. For water loss, the number of replicates ranged from 6 to 10, and for Hsp70 and respiration, four replicates were used.

### Heat shock proteins

**Fig 3:**
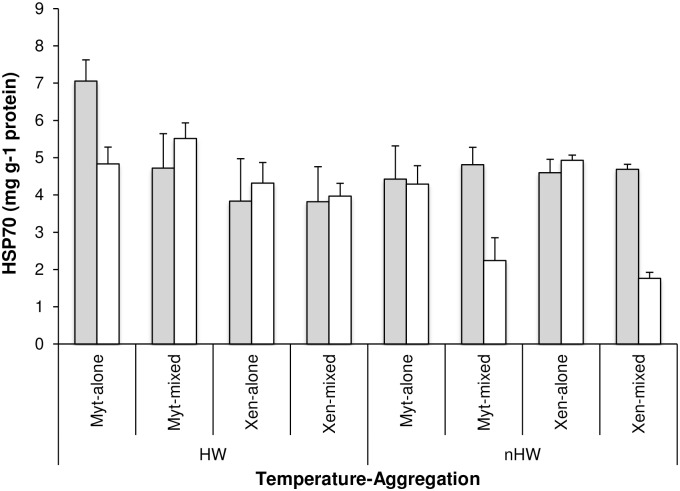
Heat shock protein content of mussels in different treatments and aggregations. Mean (+SE, n = 4) content of Hsp70 (mg g^-1^ protein) in the two temperature treatments (HW: heatwave; nHW: control), in the two types of tidal exposure (emersion in grey; re-immersion in white) and in the four aggregations (Myt-alone: monospecific *M*. *galloprovincialis*; Myt-mixed: *M*. *galloprovincialis* in mixed aggregation with *X*. *securis*, Xen-alone: monospecific *X*. *securis*; Xen-mixed: *X*. *securis* in mixed aggregation with *M*. *galloprovincialis*).

Across all treatments, the Hsp70 levels were highest in the individuals exposed to heatwave conditions (HW: 4.78 mg g^-1^ protein ± 0.29, nHW: 3.96 mg g^-1^ protein ± 0.25, p<0.05, Table 2). Nevertheless, the effect of temperature on Hsp70 levels varied depending on the tidal exposure and among aggregations (i.e. significant interaction Temperature x Tidal exposure x Aggregation, p<0.01, Table 2). After emersion for 6 h under heatwave conditions, *M*. *galloprovincialis* in monospecific aggregations showed the highest Hsp70 levels, which decreased by ~30% after re-immersion for 1.5 h (Fig 3). Moreover, Hsp70 levels of *M*. *galloprovincialis* and *X*. *securis* in mixed aggregations exposed to emersion for 6 h under control conditions, decreased by more than 50% after re-immersion for 1.5 h.

### Respiration

**Fig 4:**
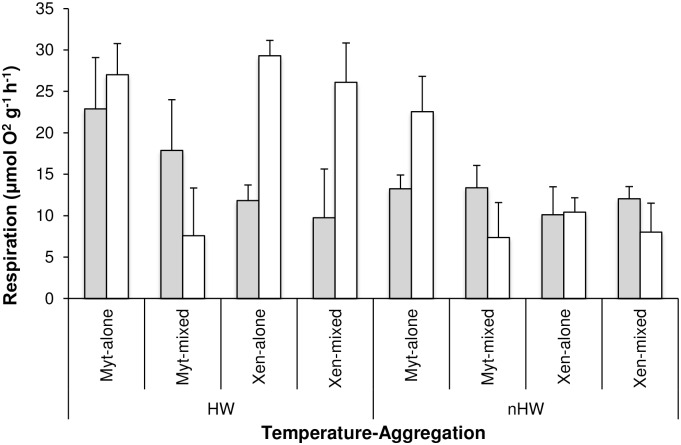
Respiration of mussels in different treatments and aggregations. Mean (+SE, n = 4) rates of respiration (μmol O_2_ h^−1^ g^−1^ dry tissue wt) in the two temperature treatments (HW: heatwave; nHW: control), in the two types of tidal exposure (emersion in grey; re-immersion in white) and in the four aggregations (Myt-alone: monospecific *M*. *galloprovincialis*; Myt-mixed: *M*. *galloprovincialis* in mixed aggregation with *X*. *securis*, Xen-alone: monospecific *X*. *securis*; Xen-mixed: *X*. *securis* in mixed aggregation with *M*. *galloprovincialis*).

Across all treatments, the respiration rates of mussels were highest under heatwave conditions (HW: 19.04 μmol O_2_ h^−1^ g^−1^ dry tissue wt ± 2.07, nHW: 12.14 μmol O_2_ h^−1^ g^−1^ dry tissue wt ± 1.24, p<0.001, Table 2). Nevertheless, the effect of temperature on respiration rates varied depending on the tidal exposure and among aggregations (i.e. significant interaction Temperature x Tidal exposure x Aggregation, p<0.05, Table 2, Fig 4). The highest rate of consumption after 6 h of emersion was observed in *M*. *galloprovincialis* exposed to heatwave conditions, especially in those individuals in monospecific aggregations (~ 23 μmol O_2_ h^−1^ g^−1^ dry tissue wt). After re-immersion, consumption of O_2_ in the mussels subjected to the heatwave conditions was still higher than after 6 h of emersion, except for *M*. *galloprovincialis* in mixed aggregations (Fig 4). The respiration rates of *X*. *securis* in both monospecific and mixed aggregations were up to 60% higher during re-immersion than after emersion (Fig 4). Under control conditions, respiration rates of mussels after emersion were similar or higher than after re-immersion, except for *M*. *galloprovincialis* in monospecific aggregations in which the opposite trend was observed.

### Gaping behaviour

**Fig 5:**
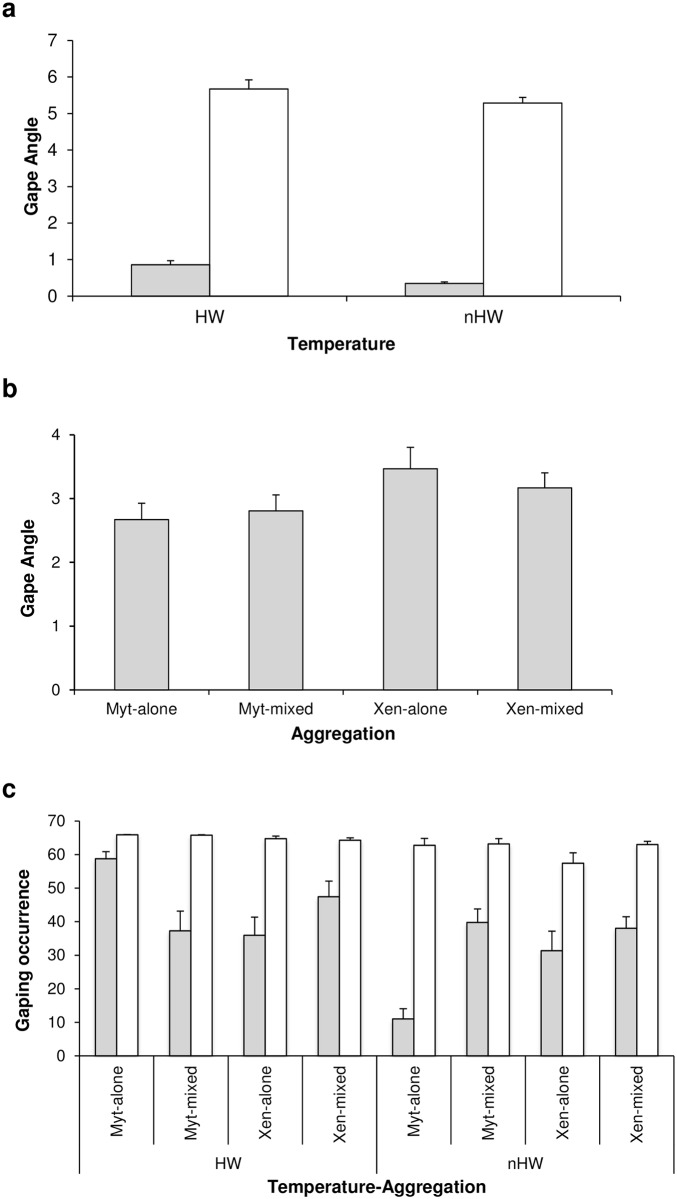
Gaping behaviour of mussels in different treatments and aggregations. Mean values (+SE) of a) gape angle in the two temperature treatments (HW: heatwave; nHW: control) and in the two types of tidal exposure (emersion in grey; immersion in white); b) gape angle in the four aggregations (Myt-alone: monospecific *M*. *galloprovincialis*; Myt-mixed: *M*. *galloprovincialis* in mixed aggregation with *X*. *securis*, Xen-alone: monospecific *X*. *securis*; Xen-mixed: *X*. *securis* in mixed aggregation with *M*. *galloprovincialis*; pooled emersion and immersion data); c) gaping occurrence in the two temperature treatments, in the two types of tidal exposure and in the four aggregations. The number of replicates varied across temperature treatments and aggregations (HW: Myt-alone = 4, Myt-mixed = 3, Xen-alone = 3, Xen-mixed = 3; nHW: Myt-alone = 4, Myt-mixed = 5; Xen-alone = 3; Xen-mixed = 6).

The angle of shell gape (aperture) was wider during immersion than emersion for *M*. *galloprovincialis* and *X*. *securis* in both monospecific and mixed aggregations (Fig 5a); however, there was a significant, almost threefold increase in the aperture of the mussels exposed to heatwave conditions during emersion (i.e. significant interaction Temperature x Tidal exposure, p<0.05, Table 3). In addition, during both emersion and immersion, the mean aperture varied significantly depending on the aggregation (Fig 5b), and the widest opening angle was observed in *X*. *securis*, especially in monospecific aggregations (p<0.05, Table 3).

**Table 3:** Summary of the results of GEEs testing the effects of experimental treatments on gaping behaviour (i.e. shell gape angle and gaping occurrence) and heart rate.

	df	χ^2^	p
*Gape angle*			
Temperature (Te)	1	3	0.106
**Tidal exposure (Ti)**	1	639	**0.000**
**Aggregation (Agg)**	3	10	**0.017**
**Te x Ti**	1	5	**0.032**
Te x Agg	3	1	0.805
Ti x Agg	3	6	0.123
Te x Ti x Agg	3	5	0.212
*Gaping occurrence*			
**Temperature (Te)**	1	4.80	**0.029**
**Tidal exposure (Ti)**	1	105.80	**0.000**
Aggregation (Agg)	3	1.90	0.589
Te x Ti	1	1.10	0.292
Te x Agg	3	6.90	0.074
**Ti x Agg**	3	12.70	**0.005**
**Te x Ti x Agg**	3	20.60	**0.000**
*Heart beat*			
Temperature (Te)	1	0.52	0.471
**Tidal exposur (Ti)**	1	5.15	**0.023**
**Aggregation (Agg)**	3	10.39	0.016
Te x Ti	1	0.10	0.752
**Te x Agg**	3	9.83	**0.021**
Ti x Agg	3	2.86	0.414
Te x Ti x Agg	3	3.26	0.353

The fixed factors included in the models were Temperature (Te), Tidal exposure (Ti) and Aggregation (Agg). The Te factor included 2 levels (HW and nHW), the Ti factor included 2 levels (emersion and immersion), and the Agg factor included 4 levels (Myt-alone, Myt-mixed, Xen-alone and Xen-mixed).

As expected, mussels were more likely to open their valves during immersion than emersion (p<0.001, Table 3). Nevertheless, the time that mussels spent with their valves open varied with the temperature treatment and depended on the tidal cycle and the aggregation (i.e. significant interaction Temperature x Tidal exposure x Aggregation, p<0.001, Table 3). During emersion, *M*. *galloprovincialis* in monospecific aggregations and *X*. *securis* in mixed aggregations were more likely to open their valves under heatwave than under control conditions. This behaviour was particularly striking in *M*. *galloprovincialis*, in which gaping occurrence increased five-fold under heatwave conditions (Fig 5c, data shown as mean gaping occurrence). However, during immersion, the gaping behaviour of both species was similar, independently of aggregation or temperature exposure.

### Heart rate

**Fig 6:**
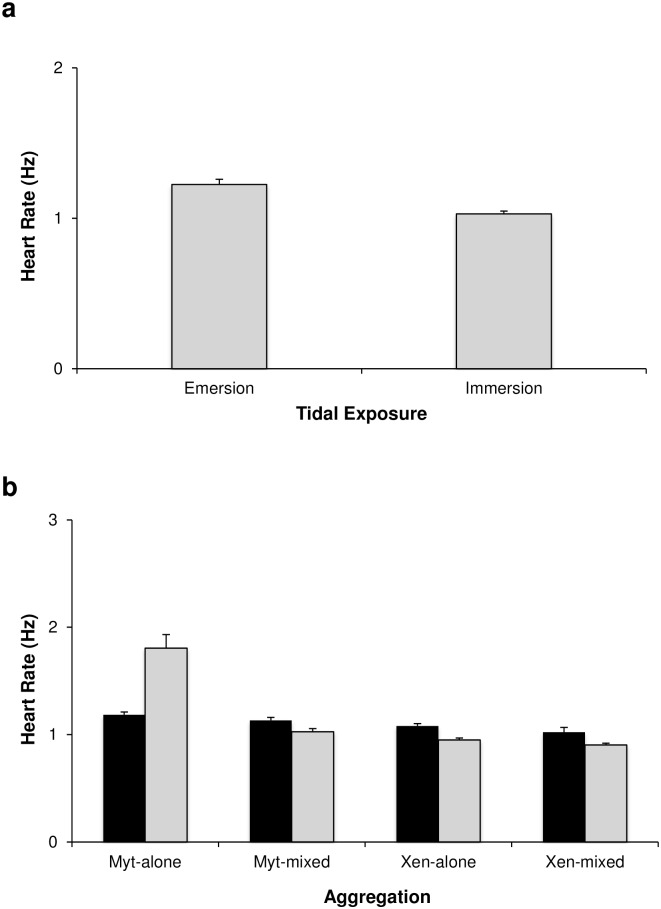
Heart rate of mussels in different treatments and aggregations. Mean (+SE) values of heart rate a) in the two types of tidal exposure (emersion and immersion); b) in the two temperature treatments (HW: heatwave in black; nHW: control in grey) and in the four aggregations (Myt-alone: monospecific *M*. *galloprovincialis*; Myt-mixed: *M*. *galloprovincialis* in mixed aggregation with *X*. *securis*, Xen-alone: monospecific *X*. *securis*; Xen-mixed: *X*. *securis* in mixed aggregation with *M*. *galloprovincialis*). The number of replicates varied across temperature treatments and aggregations (HW: Myt-alone = 4, Myt-mixed = 4, Xen-alone = 4, Xen-mixed = 4; nHW: Myt-alone = 2, Myt-mixed = 2; Xen-alone = 2; Xen-mixed = 2).

Heart rate varied significantly with tidal cycle (p<0.05, Table 3, Fig 6a), and increased during emersion. Moreover, the heart rate was affected by temperature, but the magnitude and direction of response differed among aggregations (i.e. significant interaction Temperature x Aggregation, p<0.05, Table 3). All mussels exhibited similar heart rates under both heatwave and control conditions, with a slight increase in heart rate under heat-wave conditions, except *M*. *galloprovincialis* in monospecific aggregations, in which the heart rate was much higher under control conditions and decreased by ~ 34% under heatwave conditions (Fig 6b).

## Discussion

Thermal stress and desiccation are problematical for intertidal organisms and strongly affect patterns of distribution and the dynamics of species coexistence [10,53]. This is particularly relevant when investigating interactions between native and invasive species. Differential responses of native and invasive species to stressful environmental conditions may determine the survival success of the invader and how it interacts with native species [20,24,53, 54]. For example, on the rocky shores of the Californian coast, the patterns of abundance of the native *Mytilus trossulus* (Gould 1850) and the invasive *M*. *galloprovincialis* are determined by the dynamic relationship between interspecific competition and species-specific tolerances to temperature [55].

Mussels, like many other intertidal species, use complex behavioural, physiological and biochemical mechanisms to withstand thermal stress during emersion (e.g. [9,10,15,20,24,28,41,55–59]). We found that two co-occurring intertidal mussels show different lethal and sub-lethal responses to acute thermal stress. We demonstrated gaping behavior in *M*. *galloprovincialis*, despite the species having been reported as non-gaping [24]. Consistent with the recent work of Dowd and Somero [58], we found that this species did not maintain its valves in a closed position for long periods following episodes of heat stress, although the gape angle was narrower than that of *X*. *securis*. This observation differed from that of Comeau and Babarro [40], who indicated that the gape angle was wider in *M*. *galloprovincialis* than in *X*. *securis*, although the study was conducted under different environmental conditions and mussels were always immersed. We also documented for the first time (but see [24, 60]) how species interactions modulate specific responses to environmental stress as the responses of each species varied depending on the aggregation (monospecific *versus* heterospecific).

Although mortality data may have been underestimated in this study, as we did not monitor mortality after the experimental heatwave stress period (see [58]), a clear pattern was observed. Native mussels were not able to survive the same level of environmental stress as the invasive mussels; however, the mortality of *M*. *galloprovincialis* was lower in the presence of the invader. The fact that *M*. *galloprovincialis* lost more water under the heat wave than under the control conditions (up to twice as much) may explain the higher mortality rate in monospecific aggregations. As body water plays an important role in regulating pH, death resulting from exposure to high temperatures during emersion can be attributed to an acid-base disturbance (e.g. [61]). Occasional acute temperature and desiccation stress events have been reported to cause mortality in intertidal species, including molluscs [20,22,24,62–65], although behaviour [20, 57, 58] and species interactions may influence mortality rates [24,66]. For example, survival of *M*. *galloprovincialis* was enhanced when individuals were surrounded by gaping *Perna perna* during periods of severe heat stress [20,24].

The study findings suggest that the invader ameliorated the environmental conditions experienced by *M*. *galloprovincialis* when co-occurring in aggregations. The gaping behaviour of the invader under heatwave conditions may have modified the local environment, i.e. humidity and temperature, effectively favouring *M*. *galloprovincialis* through the evaporation of water discharged during valve movements (see [24, 58]). Besides providing cooling, valve opening during episodes of elevated temperature helps to maintain oxygenation of tissues to support aerobic metabolism and prevent accumulation of metabolites [58,61,67,68], although at the cost of a greater risk of desiccation [20,24,58,59,69]. The invader gaped more during heatwave conditions than during control conditions, although the difference was not statistically significant. We hypothesize that the gaping behaviour in *X*. *securis* and particularly *M*. *galloprovincialis* during heatwave conditions was linked to higher oxygen consumption and prevention of metabolite accumulation, which is consistent with the important role of gaping in aerobic respiration [70]. Moreover, higher rates of ventilation may explain the tendency of *M*. *galloprovincialis* to exhibit greater water loss than *X*. *securis*. Further data are needed to verify this hypothesis.

The sustained valve gaping of *M*. *galloprovincialis* in monospecific aggregations when exposed to heatwave conditions was associated with a considerable increase in respiration rates during subsequent immersion. This behaviour has been observed in other gastropods such as the limpet *Patella caerulea* and in diverse porcelanid crabs, which exhibited an increased rate of O_2_ consumption following acute changes in temperature during emersion [7,71]. The enhanced rates of oxygen consumption may be caused by hyperactivity associated with excretion and the “flushing out” of ammonia and end-products of anaerobic metabolism from the body as the organism is re-immersed, i.e. oxygen debt [68]. As thermal stress has been related to oxidative stress in diverse bivalve species [72], such behaviour may also reduce oxidative stress in *M*. *galloprovincialis*, allowing macromolecular repair (see [58]). This strategy corresponds well with observations on heat shock protein expression; *M*. *galloprovincialis* in monospecific aggregations showed considerably higher production of Hsp70 during heat-wave conditions than in other treatments. Heat shock proteins comprise a complex of molecular chaperones that mitigate cellular damage and restore cellular homeostasis and are strongly indicative of stress-induced protein damage [73]. Recent evidence also suggests that expression of Hsps in *M*. *galloprovincialis* can be triggered by a reduction in the aerobic metabolic capacity induced by hypoxia [74]. It is also possible that the observed induction of Hsp70 in *M*. *galloprovincialis* was related to hypoxia rather than to thermal stress. Indeed, thermal stress and other factors such as hypoxia and desiccation may influence the production of Hsps [72]. Despite the important intraspecific variations in the expression of Hsp70, especially in *M*. *galloprovincialis*, subtle species-specific differences also occurred (but see [20]). The invader tended to produce lower levels of Hsp70 than *M*. *galloprovincialis*. The invader *X*. *securis* originates from warmer environments and Hsp70 may be induced at a higher temperature than in *M*. *galloprovincialis*. The temperature set-point for Hsp70 induction in mussels appears to vary with their thermal history [15,17,19,20,75]. Alternatively, the gaping behaviour of *X*. *securis* may diminish the risk of suffering hypoxia during emersion under heatwave conditions, and thus levels of Hsp70 expression would be lower than in *M*. *galloprovincialis*.

Although some intertidal organisms showed no differences in heart rate between emersion and immersion cycles (e.g. [76,77]), the pattern was not consistent [59,78]. In many cases, organisms showed depressed heart rate in response to thermal stress [79,80]. For example, the bay mussel *M*. *trossulus* showed a depressed heart rate in response to increasing temperature [79]. The intertidal snail *Echinolittorina malaccana* (Philippi 1847) also showed a depressed heart rate in response to warming between 30 and 45°C, which was interpreted as a strategy to conserve energy at higher temperatures [80]. In the present study, the heart rate increased overall during emersion of both species. In addition, the heart rate of *M*. *galloprovincialis* in monospecific aggregations was significantly depressed under heatwave conditions. Indeed, this species has been shown to have a remarkable capacity for metabolic depression, which is interpreted as a strategy aimed at lowering the metabolic rate, conserving energy and enhancing survival during prolonged exposure to warm air [59,78]. Surprisingly, the decreased heart rate in *M*. *galloprovincialis* was not accompanied by a reduction in gaping activity, but rather the opposite. Bradycardia (abnormally slow heart rate) may indicate cessation of oxygen uptake [61]. In our study, and specifically in the case of *M*. *galloprovincialis* in monospecific aggregations subjected to heatwave conditions, transient bradycardia during emersion may explain the elevated respiration rates subsequently recorded during immersion. It could also explain enhanced gaping activity during emersion, which is presumably an attempt to favour respiration by maintaining an appropriate O_2_ gradient across the gills and the mantle [67], and/ or an attempt to avoid oxidative stress [58]. Further experimental research aimed at directly correlating cardiac responses with other physiological variables is necessary.

In conclusion, the study findings show that the combination of direct measurements of lethal and sub-lethal responses using realistic stress provides insight into the impact of thermal disturbance on the local-scale distributions of intertidal sedentary organisms. We confirmed our initial hypothesis and showed that the invader is more resistant and resilient than *M*. *galloprovincialis* to heatwaves. We also demonstrated that *M*. *galloprovincialis* benefitted most, namely through increased resistance to thermal stress in the presence of the invader *X*. *securis*. This illustrates the complex interplay between ecological factors and physiological flexibility, which ultimately determine an organism’s fitness. Our results also highlight the potential interaction between climate change and invasive species. Both environmental conditions and the physiological response of each species to environmental stress may determine the outcome of interactions between invasive and native species in invaded habitats. Future studies addressing the role of ecological interactions in physiological responses of species will help us to gain a better understanding of invasions in the context of climate change.

## Supporting Information

S1 Fig Temperature data recorded by robo-mussels. Robo-mussels recorded temperatures in mussel beds every 30 minutes during July and August 2013.
(TIF)
