# Supplementary figures and images for: Response of Two Mytilids to a Heatwave: The Complex Interplay of Physiology, Behaviour and Ecological Interactions
Source: PLoS One. 2016 Oct 13;11(10):e0164330. doi: 10.1371/journal.pone.0164330 (PMC5063473; doi:10.1371/journal.pone.0164330)

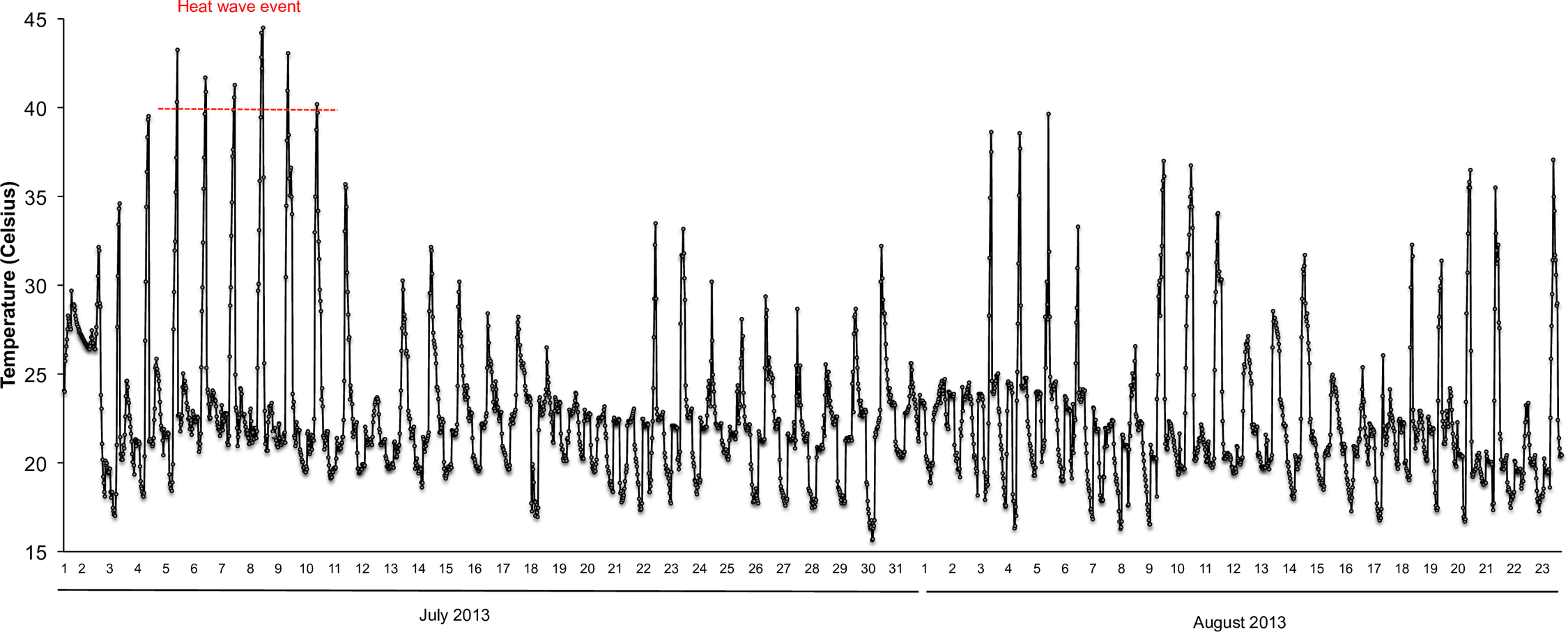

Supplement: S1 Fig — Robo-mussels recorded temperatures in mussel beds every 30 minutes during July and August 2013. (TIF) [file pone.0164330.s001.tif]
